# Epidemiological Survey of Human Dermatophytosis due to Zoophilic Species in Tehran, Iran

**Published:** 2018-12

**Authors:** Babak HASSANZADEH RAD, Seyed Jamal HASHEMI, Maryam FARASATINASAB, Javaneh ATIGHI

**Affiliations:** 1. Faculty of Veterinary Medicine, Karaj Branch, Islamic Azad University, Karaj, Iran; 2. Dept. of Medical Mycology, School of Public Health, Tehran University of Medical Sciences, Tehran, Iran; 3. Dept. of Clinical Pharmacy, Endocrine Research Center, Institute of Endocrinology and Metabolism, School of Pharmacy-International Campus, Iran University of Medical Sciences, Tehran, Iran; 4. School of Dentistry, Qazvin University of Medical Sciences, Qazvin, Iran

**Keywords:** Dermatophytosis, Dermatophyte, Zoophilic species, Anthropophilic species, *Trichophyton verrucosum*, *Microsporum Canis*

## Abstract

**Background:**

Dermatophytosis is known as one of the most frequent cutaneous infections that lead to public health problems to human and animals. The purpose of this study was to determine the prevalence of human dermatophytosis due to zoophilic species in Tehran, Iran from 2014 to 2015.

**Methods:**

Overall, 3989 patients with clinically suspected fungal infections were studied. Samples of skin, hair, and nails were examined by direct examination and culture. Direct microscopic examination was performed by KOH 15% for skin, KOH and DMSO for nail clippings and lactophenol for hair. Specimens were cultured on Sabouraud dextrose agar and mycobiotic agar.

**Results:**

Of 3989 patients, 755 (19%) suffered from dermatophytosis. Out of isolated dermatophytes, 716 (94.8%) anthropophilic, 35 (4.6%) zoophilic and 4 (0.5%) were geophilic species. Among of 35 patients with zoophilic dermatophyte infections, 65.7% were female. The most common type of zoophilic dermatophytosis according to anatomical areas was tinea manuum (34.3%) followed by tinea faciei (22.9%), tinea pedis (20%). *Trichophyton verrucosum* (57.1%) was the most commonly causative agents of zoophilic dermatophyte infections followed by *Microsporum canis* (42.9%).

**Conclusion:**

Our study showed epidemiological trends in the etiology of the agents causing dermatophytosis have changed in Tehran. Although the prevalence of zoophilic species declined in recent years, due to the tendency of most people to change lifestyles and increased urbanization, promotion of public health care and identification of new preventive and therapeutic strategies are necessary.

## Introduction

Superficial fungal infections have remained a public health problem in the world (1–5). Dermatophytosis is an infection of keratinized tissue, including the skin, hair, and nails caused by various types of dermatophyte (6). The prevalence of dermatophyte infections has been raised to more than 20%–25% of the world’s population (7).

The clinical manifestation of dermatophytosis depends on numerous factors containing pathogen species, infection site and patient’s immunological responses. Dermatophyte infections can be classified according to their host into three groups, zoophilic, geophilic, and anthropophilic. Zoophilic and geophilic species typically produce inflammatory diseases in humans, while anthropophilic species more frequently cause non-inflammatory diseases (8).

The distribution of dermatophyte infections and their causative agents have changed mainly within the last 100 yr. These changes vary in different geographic areas and are influenced by several factors including socioeconomic condition, seasonal immigration, expatriation, extreme weather, natural catastrophe, climatic factors and pharmacotherapy. Furthermore, changing lifestyle and personal hygiene are other important factors (9–11).

Although several studies have been performed on the etiology of dermatophytosis in the developing countries, epidemiological variability studies are limited over the time (10, 11). The assessment of changes in patterns of dermatophyte species could be desirable to identification of type of infection and causative agents and help in prevention and treatment of dermatophytosis in future. Zoophilic dermatophyte infections of wild and domestic animals have been known for many years. Animals are as a source for human dermatophyte infections (12,13). In Iran, the prevalence of zoophilic species has been investigated (14–24). Although zoophilic species were reported as the most common etiologic agents in the past years (14–17), anthropophilic species have been replaced in recent years (18–24).

Tehran is one of the major cities and the capital of Iran, every year is influenced by numerous factors. Therefore, the purpose of our study is to determine the prevalence pattern of human dermatophytosis due to zoophilic species and their clinical characteristic during 2014 to 2015 in Tehran, Iran.

## Materials and Methods

During Jun 2014 and Mar 2015, 3989 patients with suspected fungal infection who referred to Mycological Department of School of Public Health affiliated to Tehran University of Medical Sciences, Tehran, Iran were enrolled in this study. The local Ethics Committee of the above university approved the study protocol and informed written consent was taken from all patients.

Sampling was performed after filling out the questionnaires including age, gender, occupation, living environment, contact with animal, location and duration of the lesions. Samples of skin, hair, and nails were taken from patients by gently scraping of the affected areas. Infected areas were cleaned with 70% alcohol before sampling. Scalpels, forceps, and glass slides washed in ethanol and sterilized with a Bunsen burner were used for sampling. Direct microscopic observation was done using 15% KOH preparations for skin samples, KOH and Dimethyl sulfoxide (DMSO) for nail clippings and lactophenol alone or with heating for hair samples. Specimens were cultured on Sabouraud dextrose agar (Merck, Germany) and mycobiotic agar (Difco, East Molesey, UK) medium. Mycobiotic Agar is an excellent basal medium and antifungal agents, cycloheximide (0.5 g/L) and chloramphenicol (0.05 g/L), are added to study their effect on fungi. This medium is proven useful in the isolation of dermatophytes and other fungi from samples. Samples were incubated at 25–30 °C for 4 wk and checked every 3–4 d. The cultures were studied on the base of their macro and microscopic characteristics.

The macroscopic identification features include colony morphology, texture, growth rate, and colony pigmentation. Microscopic examination was performed with lactophenol cotton blue preparation and slide culture to test the hyphal structure, the presence, shape and arrangement of micro and macroconidia. Urease activity, pigmentation on corn meal agar with 1% dextrose, and hair perforation were applied as complementary examinations for the determination of any fungi species (22, 26).

## Results

Overall, 3989 patients with clinically suspected fungal infection, 2393 (60%) cases were male and 1596 (40%) female, with age, rang 2 months to 70 yr. Seven hundred fifty-five (19%) patients were mycological positive by direct and culture examination for different types of dermatophytosis. Out of isolated dermatophytes, 716 (94.8%) anthropophilic, 35 (4.6%) zoophilic and 4 (0.5%) were geophilic species.

Among patients with zoophilic dermatophyte infections, 65.7% were female and 34.3% were male. The highest and lowest frequency of infections was observed in patients with 41–50 (28.5%) and 51–70 (5.7%), respectively (Table 1). Tinea manuum was the most common type of dermatophytosis followed by tinea faciei, tinea pedis, tinea cruris, tinea corporis and tinea capitis (Table 2). Tinea manuum, tinea faciei, tinea pedis, tinea cruris, tinea corporis were seen more frequently in females than in males, whereas tinea capitis was only seen in males. The highest amount of tinea manuum was observed in the age group of 41–50, while tinea capitis and tinea faciei were observed in the age group of 0–10. *T. verrucosum* (57.1%) was the most commonly causative agents of zoophilic dermatophyte infections followed by *M. canis* (42.9%) (Table 3). Among 755 contaminated patients with dermatophyte infections, the prevalence of *T. verrucosum* and M. *canis* were 2.7% and 2%, respectively.

**Table 1:** Information of age and gender of patients with zoophilic dermatophyte infections

***Age Range(yr)***	***Zoophilic dermatophyte infections***		***Total (%)***
	***Male (%)***	***Female (%)***	
0–10	5 (14.2)	0 (0)	5 (14.2)
11–20	2 (5.7)	6 (17.1)	8 (22.8)
21–30	1 (2.9)	6 (17.1)	7 (20)
31–40	0 (0)	3 (8.6)	3 (8.6)
41–50	4 (11.4)	6 (17.1)	10 (28.5)
51–60	0 (0)	1 (2.9)	1 (2.9)
61–70	0 (0)	1 (2.9)	1 (2.9)

**Table 2:** Information of age range and gender of patients with zoophilic dermatophyte infections

	***Age-range(yr)***							***Gender***		***Total (%)***
	***0–10***	***11–20***	***21–30***	***31–40***	***41–50***	***51–60***	***61–70***	***M***	***F***	
T. capitis	2	0	0	0	0	0	0	2	0	2(5.7)
T. cruris	0	0	1	0	2	0	0	1	2	3(8.6)
T. corporis	0	2	1	0	0	0	0	0	3	3(8.6)
T. manium	0	3	1	2	6	0	0	3	9	12 (34.3)
T. pedis	0	2	3	0	1	1	0	3	4	7 (20)
T. faciei	3	1	1	1	1	0	1	3	5	8 (22.9)

**Table 3:** Isolated zoophilic dermatophyte species according to the clinical characteristics

**Dermatophyte sp.**	**Clinical characteristic**						
	***T. capitis***	***T. cruris(%)***	***T. corporis(%)***	***T. manuum(%)***	***T. pedis(%)***	***T. faciei(%)***	***Total (%)***
*M.canis*	1 (2.8)	0 (0)	3 (8.6%)	4 (11.4)	2 (5.7)	5 (14.3)	15 (42.8)
*T.verrucosum*	1 (2.8)	3 (8.6)	0 (0)	8 (22.9)	5 (14.3)	3 (8.6)	20 (57.2)
Total	3 (5.7)	3 (8.6)	3 (8.6)	12 (34.3)	7 (20)	8 (22.9)	35 (100)

## Discussion

Dermatophytosis is a common superficial fungal disease all over the world (1–5). Careful epidemiological study could improve our knowledge about fungal infections, their causative agent and the risk factors for infections and, has led to design of better control methods for these infection groups.

In our study, anthropophilic species were the most causative agent of dermatophytosis. These results are consistent with the observations of other studies which described a significant rise in the rate of infections caused by anthropophilic species and a reduction in the zoophilic species in different geographical area (17–24). One the possible cause of shift from zoophilic to anthropophilic species occurred over the years, could stem from genuine changes in living standards (21, 24).

In present study, dermatophytosis was generally more prevalent in men (60%) than female (40%) that similar to other researchers in Iran (24, 26). However, the majority of infected patients to zoophilic dermatophytosis were female and most of the patients were ranged 41–50 yr old. Although the dermatophytosis happens in all ages, its frequency depends on the nature of disease, climatic and occupational conditions. The higher incidence in zoophilic dermatophytosis in female could be caused by changing lifestyle such as more keeping of pets and also indirect contact with stray animal in urban areas (21, 27). In addition, promotion the levels of awareness and attitude of women towards environmental sanitation, personal hygiene resulted in more referrals to clinics and more detection of fungal infections (21).

In our observations, the most common type of zoophilic dermatophytosis was tinea manuum followed by tinea faciei, tinea pedis. Generally, the prevalence of tinea manuum is lower in different region of the world than Iran (20, 25, 27, 29–34). In Slovenia (28), Greece (29), Portugal (30), Tunisia (31), and Sweden (32), the prevalence of tinea manuum was reported 2.9%, 4.5%, 4.4%, 2% and <1% over the last decades, respectively. In Hamadan, Tehran and Mashhad tinea manuum were reported as third common type of dermatophytosis (15, 19, 24). Tinea manuum was described as the most prevalent clinical type of dermatophyte infection in Kerman (33). Different climatic condition and animal contact had an important role in the prevalence of different types of dermatophytosis. In present study, zoophilic species was only investigated which indicates more animal contact and could be caused the higher prevalence of tinea manuum. The lower incidence of tinea manuum in other counties than Iran is as a result of improved sanitary and socioeconomic conditions (29, 32). At present, training how to keep domestic animals and periodic examinations of all people in animal contact should be considered in Iran.

In our study, the lowest common type of zoophilic dermatophytosis was tinea capitis. This result confirms the finding of another recent study in Iran (34). It seems the public health care promoted, in contrast to the past in Tehran (19). Tinea capitis is a common dermatophyte infection in childhood and rarely adult (35). In our observation, all patients with this infection were child and all of them were male. Zoophilic species of dermatophytes were the main reason for tinea capitis in many parts of Iran (26, 36, 37). However, anthropophilic species was reported as the most common etiologic agents of tinea capitis in Mashhad and Tehran in recent years (24, 38, 39). In this study, we only investigated tinea capitis due to zoophilic species. We also believe a significant increase in the incidence of dermatophyte infections due to anthropophilic dermatophytes and a decrease in the zoophilic species occurred in recent years.

In present study, among 35 (4.6%) contaminated cases with zoophilic species; *T. verrucosum* was the most prevalent etiologic agent, followed by *M. canis*. On the other hand, the prevalence of *T. verrucosum* and *M. canis* were 2.7% and 2% among all of patients with dermatophytosis that agrees with the results of other studies in Iran (21, 34). *T. verrucosum* and *M. canis* are the main causative agents of dermatophyte infection in animals, and animals act as a source of human infections (26). In Iran (24, 27) *T. verrucosum* and *M. canis* are endemic in rural and urban areas, respectively. *M. canis* is the most prevalent causative agent isolated from dogs and cats. *M. canis* infection is a main epidemiologic problem in several parts of Europe (40), South America and Australia and New Zealand (21, 24). In Tehran, the most frequent etiologic agents of domestic animal dermatophytosis were *M. canis*, *T.mentagrophytes*, *T. verrucosum*, *M. gallinae*, *M. equinum*, and *M. gypseum*, respectively (41). In Tehran, *M. canis* infections have gradually increased in the past decade (21, 34, 39). During 2000–2005, the prevalence of infection was reported with M. *canis* 1.1% (39); whereas in another report (21) from 2010–2014, this prevalence has increased to 1.6%. According to our finding, the prevalence of *M. canis* was 2% during 2014–2015. This result may due to life style changes in Tehran for example, raise keeping of pets at home. However, considering religious beliefs in Iran, Iranian people keep dogs and cats as pet fewer than other countries; thus infection with *M. canis* is lower. *T. verrucosum* usually obtains from larger animal such as cattle. T. *verrucosum* was as the most commonly isolated dermatophytes in Mashhad, Hamadan, Tabriz and Isfahan cities of Iran (14, 15, 24, 42). In Tehran, the prevalence of *T. verrucosum* had significantly increased over the years 2010–2014 that indicate rural dermatophytosis spread from large animals (21). This finding could be caused by people moving from rural to urban areas.

In our observation, the most frequent form of dermatophytosis was tinea manuum. The main causative agent of tinea manuum was *T. verrucosum*. This result is in accordance with other reports in Iran (24, 33, 39). In Mashhad, zoophilic species including *T. verrucosum* and *T. mentagrophytes* were responsible for the most cases of tinea manuum (24,33). In Tehran, *T. verrucosum* was the most common species isolated from tinea manuum (39). It typically related to animal contact that transmits zoophilic species, similar to that observed in our study.

Epidemiologic features of dermatophyte infections and their etiologic agents in Iran have altered over the last decades. The fluctuations observed in the pattern of dermatophyte species involved in dermatophytosis could be due to several factors such as changing weather and climatic factors, people movements, socioeconomic and lifestyle conditions, and the introduction of new therapeutic methods. Continuous monitoring of the incidence of dermatophyte species allows the fast detection of changes in public health issues over time and improves preventive and therapeutic strategies.

## Conclusion

Although our study highlights changes in trend etiologic agents of dermatophytosis from zoophilic species to anthropophagic species; however, among zoophilic species, *T. verrucosum* and M. *canis* are still one of the important causative agents in Tehran. Therefore, promotion of public health and personal hygiene and training people who keep domestic animal for the periodic screening of dermatophytosis are necessary.

## Ethical considerations

Ethical issues (Including plagiarism, informed consent, misconduct, data fabrication and/or falsification, double publication and/or submission, redundancy, etc.) have been completely observed by the authors.
